# Conductive Education as a Method of Stroke Rehabilitation: A Single Blinded Randomised Controlled Feasibility Study

**DOI:** 10.1155/2016/5391598

**Published:** 2016-06-23

**Authors:** Judith Bek, Melanie R. Brown, Jagjeet Jutley-Neilson, Nicholas C. C. Russell, Pia A. J. Huber, Catherine M. Sackley

**Affiliations:** ^1^Faculty of Medical and Human Sciences, University of Manchester, Manchester M13 9PL, UK; ^2^National Institute of Conductive Education, Birmingham B13 3RD, UK; ^3^School of Social Sciences, Birmingham City University, Birmingham B4 7BD, UK; ^4^Faculty of Life Sciences and Medicine, King's College London, London SE1 1UL, UK

## Abstract

*Background.* Conductive Education for stroke survivors has shown promise but randomised evidence is unavailable. This study assessed the feasibility of a definitive randomised controlled trial to evaluate efficacy.* Methods.* Adult stroke survivors were recruited through local community notices. Those completing the baseline assessment were randomised using an online program and group allocation was independent. Intervention group participants received 10 weekly 1.5-hour sessions of Conductive Education at the National Institute of Conductive Education in Birmingham, UK. The control group participants attended two group meetings. The study evaluated the feasibility of recruitment procedures, delivery of the intervention, retention of participants, and appropriateness of outcome measures and data collection methods. Independent assessments included the Barthel Index, the Stroke Impact Scale, the Timed Up and Go test, and the Hospital Anxiety and Depression Scale.* Results.* Eighty-two patients were enrolled; 77 completed the baseline assessment (46 men, mean age 62.1 yrs.) and were randomised. 70 commenced the intervention (*n* = 37) or an equivalent waiting period (*n* = 33). 32/37 completed the 10-week training and 32/33 the waiting period. There were no missing items from completed questionnaires and no adverse events.* Discussion.* Recruitment, intervention, and assessment methods worked well. Transport issues for intervention and assessment appointments require review.* Conclusion.* A definitive trial is feasible. This trial is registered with ISRCTN84064492.

## 1. Introduction

Rehabilitation provision for stroke survivors is typically limited to the first few months after stroke [1, 2]. However, improvements in mobility, activities of daily living, and quality of life have been reported following rehabilitation beyond this period [3–5]. The UK Department of Health's National Stroke Strategy advised that support from stroke services should be available as required by patients and identified a need for the development of long-term community rehabilitation [6]. Similarly, the UK National Service Framework for Older People states that “rehabilitation should continue until it is clear that maximum recovery has been achieved” [7].

Conductive Education (CE) is an approach to rehabilitation that views stroke recovery as a learning process. CE was developed in Hungary in the 1940s as a specialised learning system for adults and children with neurological motor disorders [8]. Programmes are tailored to specific conditions, including stroke, Parkinson's disease, multiple sclerosis, and cerebral palsy. CE aims to help stroke survivors at any stage of recovery to maintain or increase their range and control of movement, confidence, and coordination. It teaches strategies that participants can apply to their daily activities [9]. Functional tasks are broken down into a series of components, or a “task series,” which is designed to enable participants to develop an increased awareness of their own movement and to learn the basic rules of movement solutions. Movements are practised repeatedly and rhythmically with verbal reinforcement or “rhythmical intention” and the tasks are performed in a specific order. Both repeated practice [10] and rhythmic auditory cueing [11] have previously been shown to facilitate motor learning in neurological rehabilitation. To date, there have been no randomised trials of CE for stroke. However, three small studies with pre- and post-intervention assessments have shown promise, indicating benefits in terms of motor performance, activities of daily living, and quality of life [12–14]. Caregivers have reported improvements in the individuals they cared for, as well as a decrease in their own burden [15]. However, in the absence of a control group, the specific effects of CE are yet to be demonstrated.

## 2. Methods

This study tested the feasibility and acceptability of a larger scale randomised controlled trial to assess the clinical and cost-effectiveness of a CE programme for stroke survivors versus usual care. In accordance with the recommendations by Charlesworth and colleagues [16], the following were assessed:

- The recruitment process, inclusion and exclusion criteria, and consent rate.
- The randomisation procedure and its success.
- Feasibility and acceptability of the intervention.
- Retention of patients in the study.
- Adverse events.
- Suitability and completeness of the outcome measures.

The study was a single blinded feasibility randomised controlled trial, with an intervention group and a waiting list control group. The study was carried out at the National Institute of Conductive Education in Birmingham, UK, between February 2010 and July 2012. Ethical approval for the study was obtained from the Research Governance Committee of Birmingham City Council (Ref.: 2/2/10).

Participants were recruited by centre staff through advertisement to local support groups, social care organisations, open days, and local media between February and September 2010. Adults at any stage of postacute recovery from stroke were eligible to take part in the study, provided that they were able to give informed consent and reported no current medical concerns that precluded safe participation in a rehabilitation programme. Participants were required to have a sufficient level of language comprehension to complete the questionnaires administered at baseline assessment (with assistance from a carer if required). No further inclusion or exclusion criteria were specified, with the expectation that the characteristics of the sample would reflect the broad range of individuals who might potentially benefit from a stroke rehabilitation programme. As this was a feasibility study, no formal sample size calculation was conducted, and the number of participants was restricted by the availability of funding.

Responders to the advert were invited with their carer to attend an introductory session at the National Institute for Conductive Education and were offered an initial consultation at the centre, which included an assessment of motor function and individualised goal-setting. During the consultation, centre staff assessed eligibility for the study and provided information to suitable candidates. Participants were given time to consider the information and discuss with relatives, carers, and staff. After giving informed written consent, eligible participants were randomly allocated using computer generated blocks of 10 to either immediate intervention or a waiting list control group. Randomisation was performed by an independent administrator (who was not involved in outcome assessments), using an online randomisation tool (http://www.randomization.com/), and the administrator informed patients and staff of group allocation. CE courses are run within fixed semester periods, so the exact cohort size was dependent on the number of participants enrolled by the cut-off date for course entry in each semester.

Participants were assessed by an independent assessor at baseline and after a 10-week intervention or waiting period. During the waiting period, control participants attended two introductory meetings at the CE centre, to maintain engagement in the study. Participants allocated to the control group were offered the opportunity to take part in the CE programme after reassessment.

### 2.1. Intervention

The CE programme consisted of weekly 1.5-hour sessions for 10 weeks, with up to five participants and two conductors per group. Sessions took place at the National Institute for Conductive Education in Birmingham, UK. All conductors had undergone three years of practical and theoretical training for a BA degree in Conductive Education. Partners or family members were permitted to attend and observe the session. Within each session, fine and gross motor skills were practised within task series, which were carried out in lying, sitting, and standing positions. Conductors use “rhythmical intention” to facilitate learning and action. Rhythmical intention is a link between motor and speech rhythm and is applied to each task in the programme [17]. It is a loudly stated intention, expressed in the first person singular by the participants when performing the tasks; for example, “I lift my right arm up; 1-2-3-4-5” [8]. Through verbalisation the conductor is able to present the required movement and its rhythm, which later becomes internalised as the skill becomes more automatic [18]. There are many forms of verbalisation and attention needs to be paid to the intonation and the tempo [8]. A slow rhythm is used for stroke patients, to facilitate movement without adversely influencing muscle tone [9].

### 2.2. Control Group

Participants in the waiting list group were invited to two introductory meetings during the 10-week waiting period. In the first meeting, participants watched a short film about Conductive Education and were given copies of the CE participant handbook, followed by an informal discussion session. In the second meeting, more detailed information on the CE programme was provided, and participants were given the opportunity to discuss their rehabilitation goals.

All participants had access to routine NHS care and were asked to record any rehabilitation received within the study period.

### 2.3. Outcome Measures

In preparation for a large scale trial, the primary outcome was the rating of activities of daily living (ADL). Secondary outcomes included stroke-specific quality of life, anxiety, and depression, which were assessed using self-report questionnaires, completed at each time point with assistance from carers if required. Functional mobility was independently assessed using validated performance measures.

#### 2.3.1. Activities of Daily Living and Quality of Life

The primary measure was the Barthel Index, which is a 10-item measure of self-care ADL commonly used with stroke survivors [19]. It is scored from 0 to 20, with 20 indicating independence.

The Stroke Impact Scale (SIS) is a validated stroke-specific quality of life scale, devised by stroke survivors, carers, and stroke care professionals [20]. The 59 items of the SIS (version 3.0) comprise 8 domains: strength, mobility, hand function, ADL, participation, communication, memory, and emotion. A visual analogue scale is also included, on which participants are asked to rate their recovery from stroke from 0 to 100. The SIS has been shown to be more sensitive to stroke-relevant changes than generic quality of life instruments [21, 22].

#### 2.3.2. Functional Mobility

The Timed Up and Go test (TUG) requires the participant to stand up from a chair, walk forward 3 metres, turn around, walk back, and sit down [23]. The total time taken to complete the task is recorded. The TUG has shown good test-retest reliability in stroke patients [24].

The 10-metre walking test is a measure of walking ability, typically assessing gait speed, which has been validated as a measure of functional mobility in stroke patients [25]. The participant is asked to walk at a comfortable speed along a straight 10-metre walkway, and the time taken to complete one 10-metre walk is recorded. Often, the best time out of a particular number of trials is used for analysis [24, 25]. The best-of-three measure was used in the present study, unless a participant was only able to complete one or two trials, in which case their best or only trial was used.

#### 2.3.3. Mood

Mood was assessed using the Hospital Anxiety and Depression Scale (HADS) [26], which has been shown to be appropriate for use with stroke patients living in the community [27].

### 2.4. Data Analysis

As a pilot study, analysis of the efficacy of the intervention was not appropriate. Feasibility measures and outcomes were summarised with descriptive statistics. Statistical analysis was performed using SPSS (IBM SPSS Inc., V23) software.

## 3. Results

**Figure 1:**
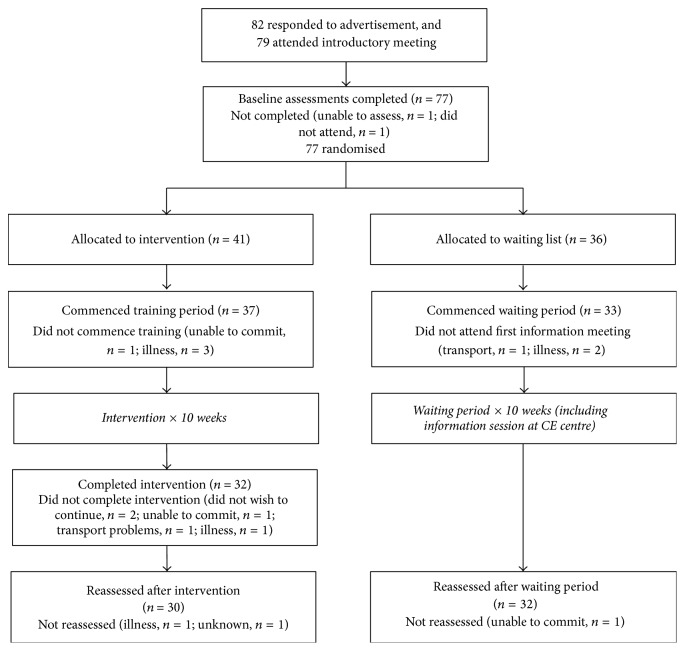
Flow of participants through the study: numbers completing each stage and reasons for non-completion.

Over an 8-month period, 82 adult stroke survivors expressed an interest in participating in the feasibility study. Seventy-nine individuals attended the introductory meeting and 77 individuals participated in the initial baseline assessment (41 in the intervention and 36 in the waiting list group); see Table 1 for baseline characteristics. Thirty-seven participants commenced the training and 32 (86%) completed the intervention. In the control group 33 participants commenced and 32 (97%) completed the waiting period. Reasons for dropping out included inability to commit (*n* = 2), illness (*n* = 1), transport problems (*n* = 1), and participants not wishing to continue because they did not feel that the CE method suited them (*n* = 2). Sixty-two participants were reassessed following the 10-week intervention period (*n* = 30) or equivalent waiting period (*n* = 32). The numbers of participants completing each stage of the study, and reasons for non-completion, are presented in Figure 1. There were no missing items from the completed outcome measures, but some questionnaires were not completed by a small number of individuals (see Table 2).  No adverse events relating to the CE programme were reported during the study.

Scores on each outcome measure at baseline (T1) and reassessment (T2) for each group are shown in Table 2. Ratings on the Stroke Impact Scale showed a greater increase in the intervention group for the domains of strength, mobility, and hand function. Participants reported mean recovery levels of 44% before and 56% after the intervention.

## 4. Discussion

The success of this randomised feasibility study suggests that a larger scale trial to evaluate the efficacy and cost-effectiveness of Conductive Education (CE) for stroke survivors is feasible.

The recruitment process worked well and stroke survivors expressed an interest in the study. The majority of individuals invited to the introductory session attended. Participants who then did not commence either the intervention or the control group meetings dropped out primarily because of transport issues or illness. Those dropping out during the intervention period reported similar problems, and some decided the commitment was too demanding. However, this was largely due to the burden of getting ready and travelling to the sessions for both the participant and carer. This would be true for any intervention delivered in a group setting and could be the focus of stroke survivor input to the development of a further trial. Missing data from the final follow-up for participants who completed the intervention could be addressed by a telephone or postal assessment. The individual outcome measures were well completed by participants with no missing items.

Feasibility studies are not designed to assess efficacy and so the between-group differences of a small study should be approached with caution. However, the results indicated some positive outcomes of the Conductive Education intervention. Improvements were demonstrated in activities of daily living and quality of life, as well as an increase in perceived recovery from stroke, relative to a waiting list control group. Ratings on the Stroke Impact Scale increased in the intervention group in the domains of strength, mobility, and hand function, and recovery from stroke was rated more highly after intervention. Importantly, there were no adverse events associated with the intervention and non-motor scores did not deteriorate. These results indicate that the chosen outcome measures were appropriate; however, the effect of the intervention on broader aspects of mood and well-being may be included in a larger study. In addition, all outcomes should be examined over a longer follow-up period to clarify the lasting effects of Conductive Education.

As a result of the inclusive approach of this study, participants spanned a broad age range and were at various stages of recovery, with a mean time after stroke of 2 years and 10 months in the intervention group. There was no systematic pattern of drop-out rates by age or time after stroke, and it was encouraging to observe that stroke survivors at any age and any stage of recovery may potentially gain benefit. Most of the participants entering the present study were more than 6 months into recovery and thus beyond the limit of the provision of statutory rehabilitation services in the UK. Conductive Education could provide an intervention for this neglected group and we suggest that a definitive randomised controlled trial would provide evidence of whether CE offers a longer-term rehabilitation option for such individuals.

The study provided encouraging results for the CE approach and demonstrated that a large scale study is feasible. Some simple practical measures could improve completion rates of the measures. It may be more difficult to unravel the problems of transport and time commitment but these issues could be explored.

## Figures and Tables

**Table 1:** Baseline demographics.

Baseline demographics	Intervention group *n* = 41	Control waiting group *n* = 36
Sex	25 M	21 M

Age	Mean 60.4 (SD = 12.6)	Mean 64.3 (SD = 13.2)
	Range 34 to 85 years	Range 36 to 88 years

Time since (first) stroke	Mean 34.5 months (SD = 39.8)	Mean 31.7 months (SD = 34.1)
	Range 3–240 months	Range 3–132 months

Barthel Index	Mean 15.9 (SD = 3.7)	Mean 14.5 (SD = 5.2)
	Range 6 to 20	Range 5 to 20

**Table 2:** Descriptive statistics for each outcome at baseline (T1) and reassessment (T2).

Outcome measure	Intervention			Control		
	*N*	T1 mean (SD)	T2 mean (SD)	*N*	T1 mean (SD)	T2 mean (SD)
Barthel Index	30	16.3 (3.3)	16.9 (3.0)	32	15.1 (4.9)	14.8 (5.0)
TUG (s)	28	34.9 (21.6)	38.0 (23.7)	27	31.5 (17.7)	47.4 (78.8)
10 m walk (s)	28	27.7 (20.5)	27.0 (20.9)	26	26.5 (16.3)	25.0 (18.8)
SIS strength	30	40.4 (24.6)	46.3 (25.3)	32	40.2 (24.7)	36.1 (20.6)
SIS memory	30	68.7 (24.5)	76.2 (22.7)	32	70.0 (22.7)	68.5 (28.4)
SIS emotion	30	63.8 (19.1)	67.4 (21.4)	31	60.5 (19.2)	61.9 (19.9)
SIS communication	30	81.6 (24.2)	83.0 (22.8)	32	63.7 (32.8)	65.0 (32.4)
SIS ADL	30	53.8 (17.7)	57.3 (20.3)	32	55.7 (23.3)	54.9 (25.1)
SIS mobility	30	57.8 (22.0)	66.8 (23.8)	32	58.5 (28.0)	58.0 (31.1)
SIS hand function	30	16.5 (23.8)	37.2 (34.6)	32	23.6 (34.0)	25.1 (30.7)
SIS participation	30	45.4 (28.9)	54.9 (27.7)	32	46.3 (27.5)	47.8 (24.2)
SIS % recovery	30	44.0 (19.5)	56.0 (21.0)	32	48.0 (18.6)	47.9 (20.0)
HADS anxiety	29	7.2 (4.4)	6.1 (3.7)	31	8.0 (5.8)	7.6 (5.5)
HADS depression	29	7.9 (3.9)	5.6 (3.8)	31	7.6 (4.6)	7.1 (4.8)

Note: Barthel Index scores out of 20; TUG: Timed Up and Go test; SIS: Stroke Impact Scale, scores out of 100; HADS: Hospital Anxiety and Depression Scale; each component scores out of 21.
